# The role of social and genetic factors in partnership trajectories and later life health

**DOI:** 10.1038/s41598-025-28752-6

**Published:** 2025-12-29

**Authors:** Meng-Jung Lin

**Affiliations:** Department of Sociology, National Taiwan University, No. 1, Sec. 4, Roosevelt Rd., Taipei, 10617 Taiwan

**Keywords:** Partnership trajectories, Sequence analysis, Polygenic scores, Life course, Aging and health, Gender differences, Diseases, Genetics, Health care, Medical research, Risk factors

## Abstract

Long-term partnership trajectories are important for understanding health inequalities in later life, yet few studies have examined how social and genetic factors jointly shape these patterns and their consequences. Using nationally representative data from the Health and Retirement Study, we applied sequence and cluster analysis to reconstruct partnership trajectories from ages 15 to 50 among 4899 non-Hispanic White respondents, identifying six distinct clusters. We then estimated how early-life socioeconomic status and polygenic scores (PGSs) for educational attainment, well-being, depressive symptoms, and body mass index were associated with trajectory membership and whether these trajectories predicted self-rated health and depressive symptoms in later life. Respondent education and the educational attainment PGS showed the most consistent associations with trajectory patterns. Stable, continuous unions were linked to better health outcomes, while never-married men reported more depressive symptoms and divorced women reported poorer self-rated health than their continuously married peers. Higher well-being PGSs and lower depressive symptom and BMI PGSs were also linked to better outcomes. These findings suggest that social and genetic selection partly but not fully account for the health advantages of stable unions. Integrating life course and genomic approaches offers new insight into the origins of health disparities in aging.

**Supplementary Information:**

The online version contains supplementary material available at 10.1038/s41598-025-28752-6.

## Introduction

Romantic partnerships play an important role in shaping health and well-being across adulthood. People in long-term, stable unions tend to report better mental and physical health than those who remain single or experience relationship disruptions such as divorce or widowhood^1–6^. These advantages are often linked to emotional support, shared economic resources, social integration, and health-related behavioral regulation^7–10^. Close relationships can buffer stress, reduce risky health behaviors, and promote routine and care-seeking, all of which may accumulate across the life course^10^. At the same time, relationship disruptions can lead to emotional distress, reduced household income, and increased caregiving demands, all of which may contribute to declines in well-being^1,11^. However, much of the existing research relies on marital status measured at a single point in time, which can miss important variation in how relationships unfold across adulthood^12,13^.

A life course perspective emphasizes that partnership histories develop over time, with earlier experiences shaping later outcomes^14,15^. Some individuals remain in stable, long-term unions, whereas others experience repeated transitions or remain unpartnered for extended periods. Such patterns may reflect structural conditions such as childhood disadvantage or lower parental education^11,16,17^ as well as individual factors such as genetic predispositions and personality that influence relationship behaviors and expectations. These factors can, in turn, affect health outcomes later in life. For example, recent research has shown that fertility history and partnership trajectories are linked to cognitive outcomes in later life, with single and childless individuals reporting lower cognitive functioning^18^.

Genetic factors are another potential source of variation in partnership behaviors. Genome-wide association studies have identified genetic variants associated with traits such as reproductive timing^19–22^, educational attainment^23,24^, well-being, and depression^25–27^, all of which may be related to long-term relationship patterns. Polygenic scores (PGSs) summarize these associations by aggregating the effects of many variants into a single score that reflects an individual’s inherited propensity for a trait. For example, a PGS for depression estimates genetic liability to depressive symptoms, whereas a PGS for educational attainment may be related to long-term planning. Incorporating PGSs alongside social background variables may thus provide a more comprehensive understanding of the factors shaping partnership trajectories and their links to later‑life health.

Recent sociogenomic studies have begun to examine genetic influences on relationship outcomes more directly. Using data from the Norwegian Mother, Father and Child Cohort Study (MoBa), Jørgensen et al.^28^ reported that polygenic indices for traits such as educational attainment, depression, subjective well-being, and neuroticism were associated with the risk of partnership dissolution, with some associations persisting after adjusting for shared family background. Gueltzow et al.^29^ analyzed data from Finnish adults in the FINRISK and Health 2000 and 2011 surveys and found that both genetic propensity to depression and partnership status were associated with antidepressant use, though partnership did not moderate genetic risk. Evidence from Domingue et al.^30^, based on the U.S. Health and Retirement Study (HRS), further demonstrates that individuals with higher well‑being PGSs exhibit smaller increases in depressive symptoms following spousal bereavement, illustrating how genetic predispositions can shape responses to major life stressors. These studies underscore the value of combining genomic and social data to study family and partnership dynamics.

Building on this evidence, our study incorporates PGSs for educational attainment, BMI, well‑being, and depressive symptoms. Educational attainment is widely recognized in demographic and sociological research as a key predictor of union formation and stability^31,32^, and its PGS may reflect inherited advantages relevant to relationship trajectories. Evidence of genetic assortative mating on education in the U.S.^33^ highlights how inherited propensities related to education can shape partnership patterns. In addition, genetic propensities for psychological resilience or vulnerability may also influence both partnership stability and later-life mental health^30^. Two PGSs are therefore considered in the study. The well-being PGS is based on measures of subjective well-being that capture life satisfaction, positive affect, and happiness^26^, whereas the depressive symptoms PGS reflects genetic liability toward negative affect and mood disturbance^30^.

Gender is also a key factor in how partnership trajectories are shaped and how they affect health. Men often receive more consistent health benefits from long term unions, partly because women disproportionately take on emotional and caregiving responsibilities^5,34^. For women, particularly after divorce or widowhood, the cumulative effects of caregiving, stress, and financial strain can reduce the potential benefits of marriage^1,35,36^. Consistent with these patterns, a study finds that individuals in stable cohabiting unions report higher life satisfaction and lower depressive symptoms than those who are single or in unstable relationships^37^. Their results also highlight gender differences: men who remain single long term have poorer well-being, whereas women who experience repeated relationship instability report greater distress. Recent evidence further shows that gender differences in social isolation vary across the life course and depend on partnership history. Men are more socially isolated in adolescence and early adulthood, whereas women become more isolated in later life, particularly after relationship disruptions^38^. These patterns may have important implications for health in later life.

In this study, we analyze self-rated health separately as an indicator of overall health^39^. We also adopt a subjective well-being framework distinguishing evaluative life satisfaction, affective feelings, and eudaimonic purpose and meaning^40,41^, and we focus on the affective component, specifically negative affect, measured as depressive symptomatology using the CESD scale^42,43^. Drawing on nationally representative longitudinal data from the Health and Retirement Study (HRS), a survey of U.S. adults aged 50 and above, we address three questions. First, which social and genetic characteristics are associated with distinct partnership trajectories across adulthood? Second, how are these trajectories related to self-rated health and depressive symptoms in later life after accounting for genetic predispositions? Third, do these associations differ between men and women? Using sequence and cluster analysis, we identify distinct partnership trajectories, examine their associations with later-life health outcomes, and assess whether these patterns vary by gender. By combining life course theory with molecular genetic data, this study provides new evidence on the social and biological pathways that contribute to health inequalities in older adulthood.

## Results

### Partnership trajectory sequences and clusters

**Fig. 1:**
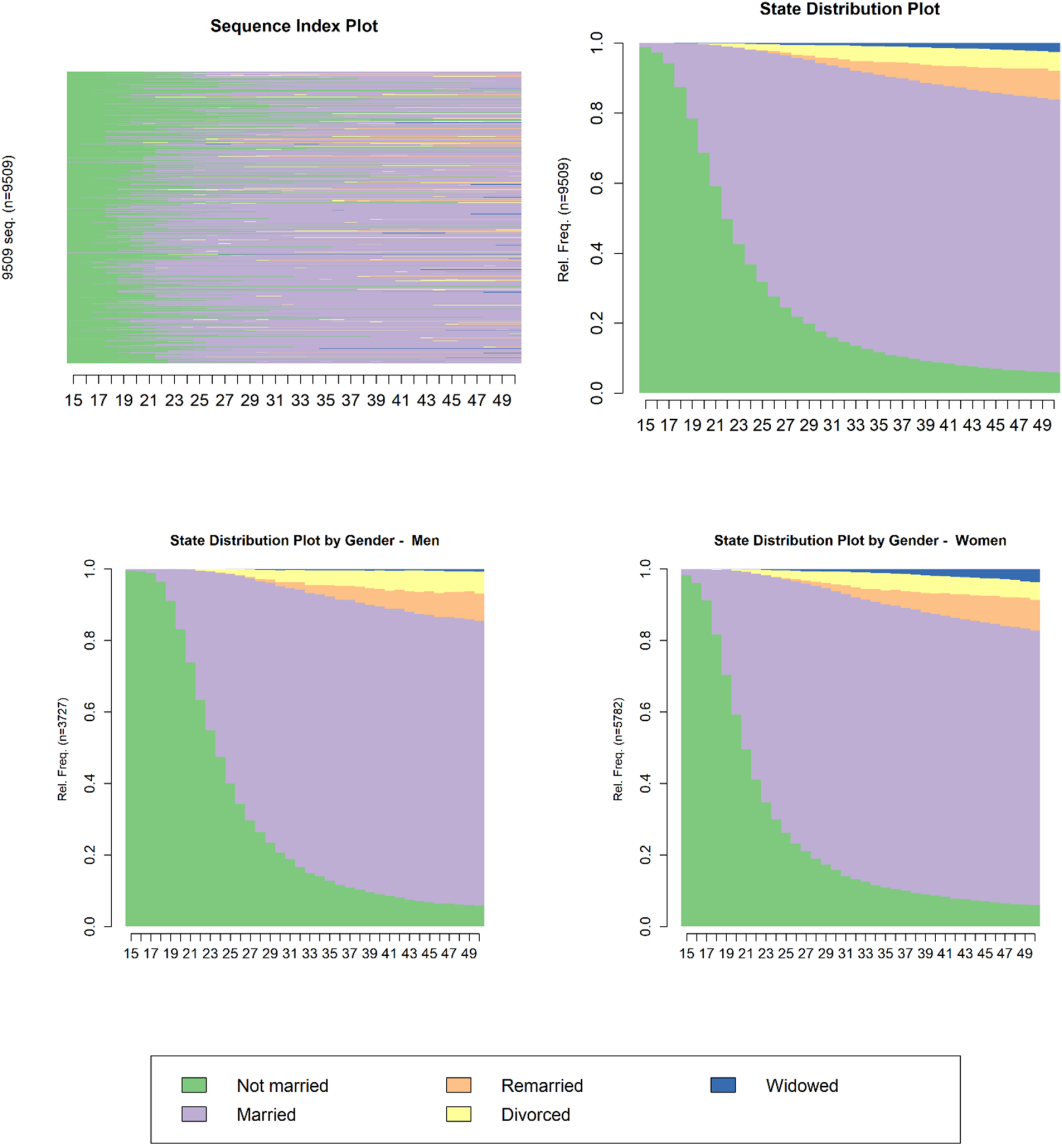
Partnership trajectories from ages 15 to 50 and state distributions by gender. The sequence index plot (top left) shows individual partnership trajectories between ages 15 and 50. The state distribution plot (top right) depicts the overall proportion of respondents in each partnership state by age. The lower panels show state distributions separately for men (bottom left) and women (bottom right). Colors represent different partnership states: not married (green), married (purple), remarried (orange), divorced (yellow), and widowed (blue).

Figure 1 summarizes individual partnership state sequences from ages 15 to 50. The sequence index plot (top left) shows that most respondents follow a “single to married” pattern, with some experiencing divorce or widowhood later in life. The state distribution plot (top right) displays the overall proportion of respondents in each partnership state at each age. The lower panels present state distributions separately for men and women, indicating that women tend to enter marriage slightly earlier than men. On average, men and women spend similar amounts of time in each state, with most years spent married and relatively few years spent divorced, remarried, or widowed.

**Fig. 2:**
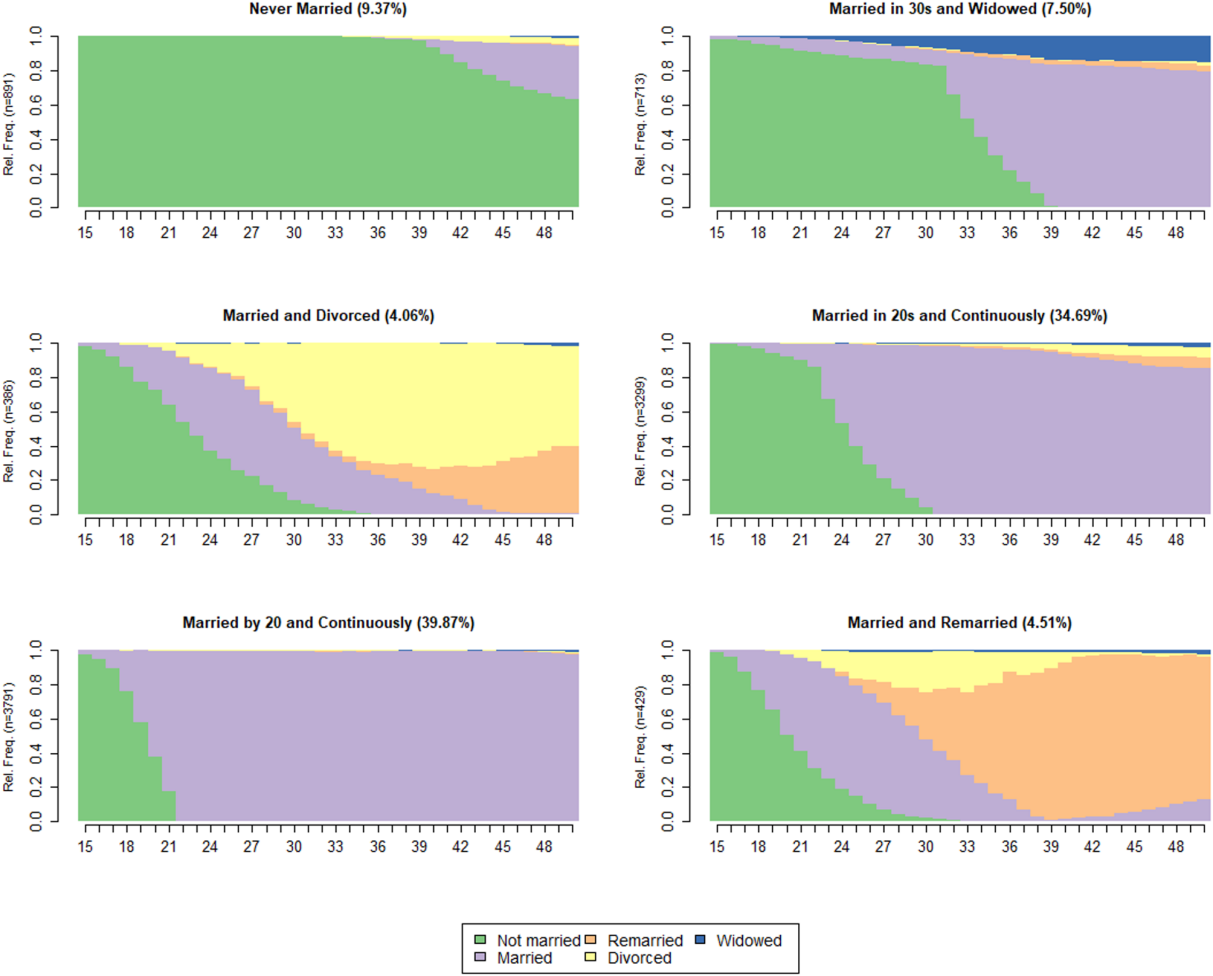
State distributions of partnership trajectories from ages 15 to 50 by cluster. Each panel shows the proportion of respondents in each partnership state (not married, married, remarried, divorced, widowed) at each age, with sample sizes for each cluster shown in parentheses on the y-axis. Percentages of respondents belonging to each cluster are reported next to the cluster names. The six clusters are Never Married, Married in 30s and Widowed, Married and Divorced, Married in 20s and Continuously Married, Married by 20 and Continuously Married, and Married and Remarried.

The clustering analysis identified six distinct partnership trajectory clusters. The average silhouette width for this solution (ASW = 0.4518) indicates a reasonable balance between within‑group similarity and between‑group separation. The R^2^ value (0.6246) shows that the solution explains a substantial proportion of variance, and the Calinski–Harabasz index (CH = 3162.5) further supports the quality of the clustering. Hubert’s Gamma statistic (HG = 0.8131) also indicates strong agreement between the distance matrix and the cluster assignments, and the relatively low hierarchical complexity (HC = 0.0646) suggests that the clusters are interpretable. As shown in Fig. 2, the six clusters represent distinct and substantively meaningful partnership trajectories. Labeling draws on state distributions and on the mean and median age at first marriage for each cluster (Supplementary Table S1). The clusters are named as Never Married, Married in 30s and Widowed, Married and Divorced, Married in 20s and Continuously Married, Married by 20 and Continuously Married, and Married and Remarried.

### Descriptive statistics

Table 1 presents descriptive statistics for the analytic sample of non‑Hispanic White respondents in the HRS. Among the six trajectory clusters, 44.2% of respondents are in the Married by 20 and Continuously cluster, 36.0% are in the Married in 20s and Continuously Married cluster, 5.7% are Never Married, 5.5% are Married in 30s and Widowed, 4.8% are Married and Remarried, and 3.7% are Married and Divorced. The distribution differs by gender: 52.8% of women are in the Married by 20 and Continuously cluster compared with 31.4% of men, whereas 45.4% of men are in the Married in 20s and Continuously Married cluster compared with 29.7% of women.

**Table 1:** Descriptive statistics of the analytic sample (non-Hispanic White respondents).

	Non-Hispanic Whites					
	All (N = 4899)		Male (N = 1962)		Female (N = 2937)	
Variable	Mean	S.D	Mean	S.D	Mean	S.D
Health outcomes						
Self-rated health	3.801	1.004	3.805	1.004	3.798	1.005
CESD score	0.978	1.621	0.782	1.401	1.109	1.740
Partnership trajectory						
Never married	0.057	–	0.066	–	0.051	–
Married in 30s and widowed	0.055	–	0.076	–	0.041	–
Married and divorced	0.037	–	0.045	–	0.032	–
Married in 20s and continuously married	0.360	–	0.454	–	0.297	–
Married by 20 and continuously married	0.442	–	0.314	–	0.528	–
Married and remarried	0.048	–	0.045	–	0.050	–
Polygenic scores						
Educational attainment PGS	0.074	0.988	0.078	1.009	0.072	0.974
Well-being PGS	0.011	0.993	0.029	0.980	− 0.001	1.002
Depressive symptoms PGS	− 0.039	1.015	− 0.055	0.994	− 0.028	1.028
BMI PGS	− 0.034	0.999	− 0.072	0.991	− 0.009	1.004
Socioeconomic status						
Years of education	13.812	2.295	14.136	2.345	13.595	2.235
Mother’s education	11.161	2.934	11.415	2.880	10.990	2.957
Father’s education	10.859	3.495	11.085	3.485	10.709	3.494
Childhood SES						
Poor	0.212	–	0.221	–	0.206	–
Average	0.689	–	0.682	–	0.694	–
Well off	0.086	–	0.084	–	0.087	–
Varied or missing	0.013	–	0.013	–	0.014	–
Self-reported gender						
Male	0.400	–	–	–	–	–
Female	0.600	–	–	–	–	–
Birth year	1944.329	9.890	1944.400	9.550	1944.282	10.112
Birth cohort						
Cohorts 0–3; < 1924 to 1947	0.422	–	0.425	–	0.421	–
Cohorts 4 & 5: 1948 to 1959	0.362	–	0.365	–	0.360	–
Cohorts 6 & 7: 1960 to 1971	0.215	–	0.210	–	0.219	–
Age difference between outcome measured and age 50						
Age difference: self-rated health	4.911	5.415	5.084	4.962	4.796	5.695
Age difference: CESD	5.689	5.619	6.025	5.331	5.465	5.794

Means and standard deviations (SD) for all variables used in the analyses are presented for the pooled sample and stratified by gender.

CESD, Center for Epidemiologic Studies Depression scale; PGS, Polygenic score; Childhood SES, Childhood socioeconomic status.

Regarding well‑being, the mean self‑rated health score is 3.80. Women report similar self‑rated health (3.80) compared with men (3.81) but have higher CESD scores (1.11 vs. 0.78), indicating more depressive symptoms on average.

All polygenic scores are standardized to have a mean close to zero and a standard deviation near one. Minor deviations from zero reflect the restriction of the analytic sample to respondents with complete partnership histories and well‑being data.

Respondents report an average of 13.81 years of education, and both mothers and fathers have an average of approximately 11 years of education. Childhood socioeconomic status (childhood SES) also varies: 68.9% reported that their family’s financial situation at age 16 was “average”, 21.2% “poor”, and 8.6% “well off”. The analytic sample is 60.0% female, and the mean birth year is 1944.33.

### Social and genetic characteristics associated with partnership trajectories

Table 2 presents results from multinomial logistic models predicting partnership trajectory clusters relative to the Married in 20s and Continuously Married cluster. The models include socioeconomic background variables and four polygenic scores: educational attainment, well-being, depressive symptoms, and BMI.

**Table 2:** Multinomial logistic models for partnership trajectory clusters among non-Hispanic White respondents (reference = married in 20s and continuously married).

	All					Male					Female				
	Never Married	Married in 30s and widowed	Married and divorced	Married by 20 and continuously	Married and remarried	Never Married	Married in 30s and widowed	Married and divorced	Married by 20 and continuously	Married and remarried	Never Married	Married in 30s and widowed	Married and divorced	Married by 20 and continuously	Married and remarried
Variables	Coef (S.E.)	Coef (S.E.)	Coef (S.E.)	Coef (S.E.)	Coef (S.E.)	Coef (S.E.)	Coef (S.E.)	Coef (S.E.)	Coef (S.E.)	Coef (S.E.)	Coef (S.E.)	Coef (S.E.)	Coef (S.E.)	Coef (S.E.)	Coef (S.E.)
Polygenic scores															
Educational attainment PGS	0.045	0.128	− 0.099	− 0.088*	− 0.249**	0.052	0.161	0.058	− 0.021	− 0.393**	0.020	0.082	− 0.245*	− 0.138**	− 0.187
	(0.073)	(0.075)	(0.089)	(0.039)	(0.079)	(0.108)	(0.102)	(0.129)	(0.060)	(0.130)	(0.102)	(0.112)	(0.124)	(0.051)	(0.102)
Well-being PGS	− 0.128	− 0.039	0.092	0.043	0.033	− 0.248*	− 0.181	0.053	0.009	0.109	− 0.042	0.107	0.111	0.077	0.010
	(0.072)	(0.073)	(0.087)	(0.038)	(0.078)	(0.109)	(0.103)	(0.128)	(0.061)	(0.129)	(0.097)	(0.107)	(0.122)	(0.049)	(0.098)
Depressive symptoms PGS	− 0.054	0.004	0.123	0.065	− 0.082	− 0.013	− 0.051	0.173	0.138*	− 0.010	− 0.091	0.062	0.092	0.026	− 0.125
	(0.071)	(0.073)	(0.087)	(0.037)	(0.077)	(0.107)	(0.101)	(0.128)	(0.062)	(0.129)	(0.094)	(0.106)	(0.121)	(0.048)	(0.097)
BMI PGS	− 0.031	− 0.110	− 0.030	− 0.004	0.019	− 0.009	− 0.241*	0.135	0.130*	0.100	− 0.080	0.025	− 0.189	− 0.081	− 0.018
	(0.074)	(0.075)	(0.090)	(0.039)	(0.080)	(0.111)	(0.105)	(0.133)	(0.064)	(0.137)	(0.101)	(0.110)	(0.126)	(0.051)	(0.102)
Socioeconomic status															
Years of education	− 0.039	− 0.049	− 0.096*	− 0.220***	− 0.156***	− 0.053	− 0.026	− 0.085	− 0.165***	− 0.078	− 0.009	− 0.066	− 0.109	− 0.261***	− 0.216***
	(0.034)	(0.035)	(0.041)	(0.018)	(0.036)	(0.050)	(0.047)	(0.058)	(0.026)	(0.058)	(0.048)	(0.052)	(0.060)	(0.024)	(0.048)
Mother’s education	− 0.012	− 0.005	− 0.066	− 0.006	0.003	− 0.017	0.003	− 0.054	− 0.005	− 0.036	− 0.015	− 0.013	− 0.079	− 0.005	0.025
	(0.030)	(0.031)	(0.037)	(0.015)	(0.033)	(0.046)	(0.043)	(0.056)	(0.025)	(0.056)	(0.040)	(0.044)	(0.051)	(0.020)	(0.042)
Father’s education	0.019	0.028	0.024	− 0.009	0.013	0.043	0.059	0.055	− 0.045*	0.003	0.007	0.004	0.006	0.012	0.024
	(0.025)	(0.026)	(0.031)	(0.013)	(0.028)	(0.039)	(0.037)	(0.047)	(0.021)	(0.046)	(0.034)	(0.037)	(0.042)	(0.017)	(0.035)
Childhood SES (Ref. = Poor)															
Average	− 0.205	− 0.108	− 0.424*	− 0.005	0.054	− 0.160	− 0.084	− 0.520	0.026	0.481	− 0.319	− 0.228	− 0.348	− 0.051	− 0.169
	(0.167)	(0.176)	(0.197)	(0.087)	(0.189)	(0.256)	(0.248)	(0.273)	(0.135)	(0.342)	(0.224)	(0.253)	(0.290)	(0.117)	(0.233)
Well off	− 0.192	− 0.225	0.007	− 0.188	0.156	− 0.113	− 0.287	− 0.845	− 0.159	0.429	− 0.378	− 0.315	0.513	− 0.201	0.014
	(0.262)	(0.276)	(0.297)	(0.148)	(0.295)	(0.394)	(0.388)	(0.516)	(0.251)	(0.518)	(0.359)	(0.401)	(0.391)	(0.189)	(0.366)
Varied or missing	− 0.271	0.373	− 0.324	0.154	− 0.282	0.495	0.650	− 0.428	− 0.069	− 12.573	− 0.993	0.012	− 0.382	0.214	− 0.087
	(0.637)	(0.524)	(0.768)	(0.301)	(0.765)	(0.818)	(0.705)	(1.086)	(0.503)	(722.899)	(1.068)	(0.812)	(1.100)	(0.393)	(0.805)
Female	0.111	− 0.248	0.005	0.910***	0.481***										
	(0.131)	(0.133)	(0.159)	(0.071)	(0.145)										
Birth year	0.063***	0.058***	0.093***	0.004	0.066***	0.094***	0.063**	0.105***	0.010	0.078**	0.051***	0.058***	0.102***	0.000	0.059***
	(0.012)	(0.013)	(0.014)	(0.007)	(0.013)	(0.022)	(0.022)	(0.023)	(0.013)	(0.029)	(0.015)	(0.016)	(0.019)	(0.009)	(0.015)
Birth cohort (Ref. = Cohorts 0–3; < 1924 to 1947)															
Cohorts 4 and 5: 1948 to 1959	− 0.363	− 0.419	0.384	− 0.049	0.222	− 0.339	− 0.448	− 0.372	− 0.042	0.576	− 0.547*	− 0.447	1.110**	− 0.090	0.040
	(0.207)	(0.214)	(0.240)	(0.116)	(0.223)	(0.341)	(0.346)	(0.373)	(0.197)	(0.450)	(0.269)	(0.285)	(0.375)	(0.147)	(0.264)
Cohorts 6 and 7: 1960 to 1971	− 0.382	− 0.404	− 0.519	− 0.681***	− 0.224	− 0.439	− 0.200	− 0.949	− 0.650*	0.319	− 0.574	− 0.669	− 0.197	− 0.756***	− 0.485
	(0.287)	(0.303)	(0.327)	(0.183)	(0.315)	(0.487)	(0.511)	(0.538)	(0.319)	(0.649)	(0.366)	(0.388)	(0.460)	(0.227)	(0.370)
Constant	− 124.632***	− 113.501***	− 181.547***	− 4.091	− 128.839***	− 184.264***	− 123.840**	− 205.301***	− 17.019	− 153.869**	− 99.791***	− 113.010***	− 198.936***	3.895	− 113.037***
	(23.413)	(24.716)	(26.559)	(13.751)	(25.567)	(41.965)	(43.485)	(45.410)	(24.491)	(55.213)	(28.750)	(30.429)	(37.543)	(16.944)	(29.013)
Observations	4922					1965					2957				
Pseudo R-squared	0.0828					0.0794					0.0748				
(− 2) Log-likelihood	11,950					4982					6822				
Chi-squared	1078					429.8					551.9				

Standard errors in parentheses.

PGS, Polygenic score; Childhood SES, Childhood socioeconomic status. Top ten PCs are controlled in the models.

***p < 0.001; **p < 0.01; *p < 0.05.

Among the polygenic scores, the educational attainment PGS shows the most consistent associations. It is negatively associated with the Married by 20 and Continuously ($\beta = - 0.09, SE = 0.04, p = 0.022$) and Married and Remarried ($\beta = - 0.25, SE = 0.08, p = 0.002$) clusters in the pooled sample. In gender‑stratified models, a higher educational attainment PGS is also negatively associated with the Married and Remarried cluster for men ($\beta = - 0.39, SE = 0.13, p = 0.002$). For women, it is negatively associated with the Married and Divorced ($\beta = - 0.25, SE = 0.12, p = 0.048$) and Married by 20 and Continuously ($\beta = - 0.14, SE = 0.05, p = 0.007$) clusters. The other polygenic scores show more limited associations. Among men, the well‑being PGS is negatively associated with the Never Married cluster ($\beta = - 0.25, SE = 0.11, p = 0.024$), and the depressive symptoms PGS is positively associated with the Married by 20 and Continuously cluster ($\beta = 0.14, SE = 0.06, p = 0.026$). The BMI PGS is negatively associated with the Married in 30s and Widowed cluster ($\beta = - 0.24, SE = 0.11, p = 0.022$) and positively associated with the Married by 20 and Continuously cluster for men ($\beta = 0.13, SE = 0.06, p = 0.041$). None of the health‑related PGSs are significantly associated with partnership trajectories among women.

Social factors remain strongly associated with partnership trajectories after accounting for genetic predispositions. More years of education are linked to a lower likelihood of being in the Married and Divorced ($\beta = - 0.10, SE = 0.04, p = 0.019$), Married by 20 and Continuously ($\beta = - 0.22, SE = 0.02, p < 0.001$), or Married and Remarried ($\beta = - 0.16, SE = 0.04, p < 0.001$) clusters compared with the reference group. The negative association with the Married and Remarried cluster is particularly evident among women ($\beta = - 0.22, SE = 0.48, p < 0.001$). Father’s education is negatively associated with the Married by 20 and Continuously cluster for men ($\beta = - 0.05, SE = 0.02, p = 0.030$). In addition, respondents who reported average SES at age 16, compared with those from poor backgrounds, are less likely to be in the Married and Divorced cluster ($\beta = - 0.43, SE = 0.20, p = 0.031$).

Gender and cohort differences are also apparent. Women are more likely than men to be in the Married by 20 and Continuously ($\beta = 0.91, SE = 0.07, p < 0.001$) and Married and Remarried ($\beta = 0.48, SE = 0.15, p = 0.001$) clusters. Compared with the oldest cohort (born < 1924–1947), the youngest cohort (born 1960–1971) is less likely to be in the Married by 20 and Continuously cluster ($\beta = - 0.68, SE = 0.18, p < 0.001$). Among women, the middle cohort (born 1948–1959) is less likely to be Never Married but more likely to be in the Married and Divorced cluster ($\beta = 1.11, SE = 0.38, p = 0.003$).

Overall, both social factors and polygenic scores are associated with partnership trajectories. Respondent education and the educational attainment PGS show the clearest and most consistent associations across clusters, whereas the effects of the well‑being, depressive symptoms, and BMI PGSs are more limited and largely specific to men. Parental education and childhood socioeconomic status also play a role, but their associations are weaker and less consistent.

### Associations of partnership trajectories with health outcomes

#### Self-rated health

**Fig. 3:**
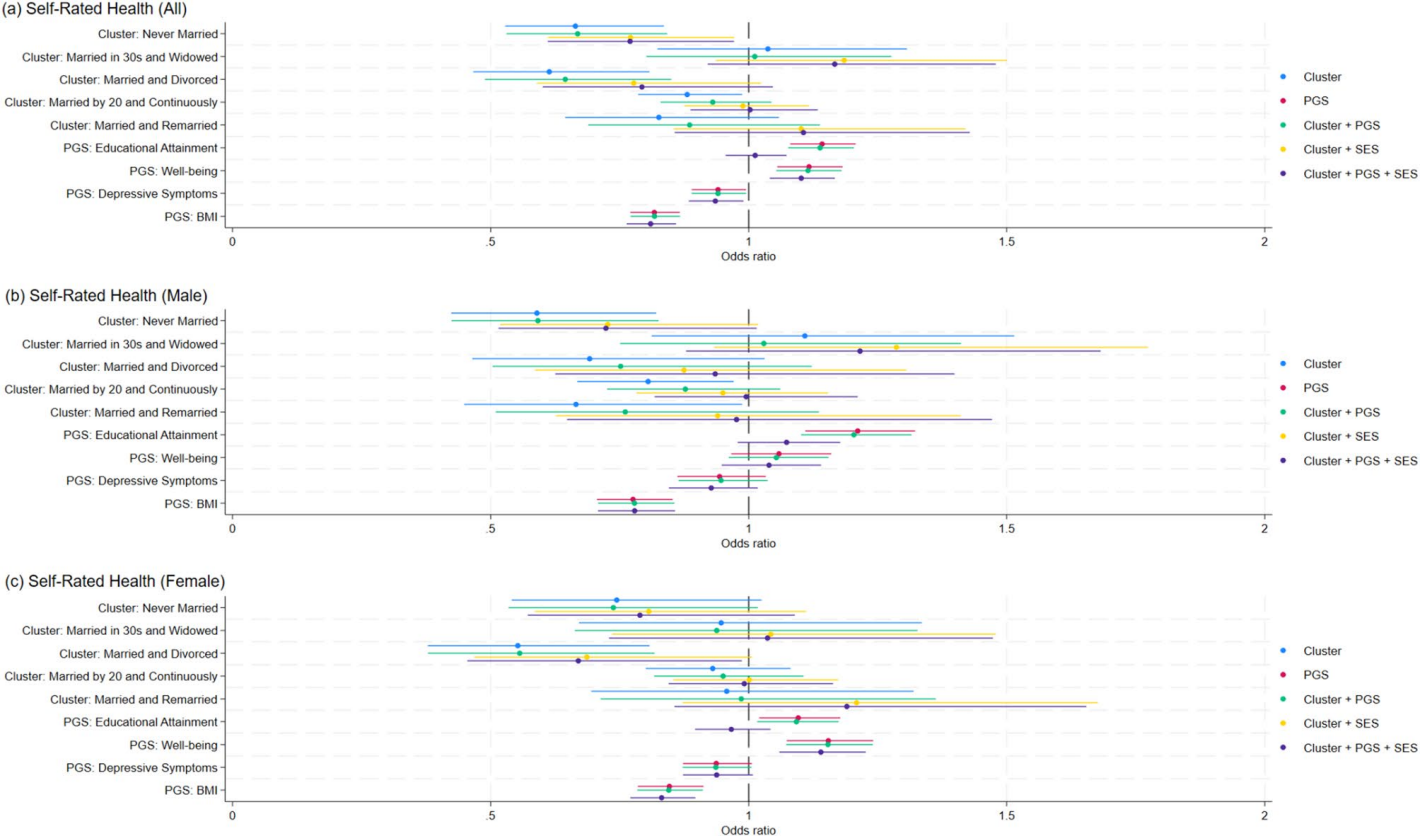
Odds ratios for Self-rated Health associated with partnership trajectories and polygenic scores (overall and by gender). Odds ratios and 95% confidence intervals from ordered logit models predicting Self-rated Health. Results are shown for the pooled sample (**a**), men (**b**), and women (**c**). Each set of points represents estimates from models including different combinations of predictors: partnership trajectory clusters only, polygenic scores (PGS) only, clusters plus PGS, clusters plus socioeconomic status (SES) and control variables, and clusters plus PGS, SES, and controls. The reference group for partnership clusters is “Married in 20s and Continuously Married”.

Figure 3 shows that when only partnership trajectories are included, respondents in the Never Married, Married and Divorced, and Married by 20 and Continuously clusters report poorer health compared with those in the Married in 20s and Continuously Married cluster, with the largest difference for the Married and Divorced cluster. The coefficient for the Married by 20 and Continuously cluster becomes insignificant after PGSs are added. Associations for the other clusters weaken further as SES and control variables are included (see Supplementary Table S4).

In the fully adjusted model (Table 3, Panel A), two clusters remain significantly associated with self‑rated health. Respondents in the Never Married cluster ($\beta = - 0.26, SE = 0.12, p = 0.027$) report poorer health in the pooled sample, and among women, those in the Married and Divorced cluster ($\beta = - 0.4, SE = 0.20, p = 0.043$) report poorer health compared with the reference group.

**Table 3:** Ordered logit models for self-rated health (Panel A) and negative binomial models for CESD score (Panel B) among non-Hispanic White respondents.

	A. Self-rated health			B. CESD score		
	All	Male	Female	All	Male	Female
Variables	Coef (S.E.)	Coef (S.E.)	Coef (S.E.)	Coef (S.E.)	Coef (S.E.)	Coef (S.E.)
Partnership trajectory (Ref. = Married in 20s and continuously married)						
Never married	− 0.261*	− 0.324	− 0.236	0.237*	0.334*	0.144
	(0.118)	(0.173)	(0.164)	(0.103)	(0.157)	(0.137)
Married in 30s and widowed	0.154	0.195	0.036	0.066	0.116	− 0.025
	(0.121)	(0.166)	(0.179)	(0.109)	(0.154)	(0.155)
Married and divorced	− 0.232	− 0.067	− 0.400*	0.141	0.258	0.065
	(0.142)	(0.205)	(0.198)	(0.125)	(0.187)	(0.169)
Married by 20 and continuously married	0.003	− 0.005	− 0.009	− 0.030	− 0.133	0.018
	(0.063)	(0.100)	(0.082)	(0.057)	(0.097)	(0.070)
Married and remarried	0.101	− 0.024	0.174	0.035	− 0.364^a^	0.212^a^
	(0.130)	(0.209)	(0.168)	(0.113)	(0.207)	(0.136)
Polygenic scores						
Educational attainment PGS	0.012	0.071	− 0.034	0.018	− 0.000	0.038
	(0.030)	(0.047)	(0.039)	(0.026)	(0.042)	(0.034)
Well-being PGS	0.097***	0.039	0.131***	− 0.071**	− 0.055	− 0.078*
	(0.029)	(0.047)	(0.037)	(0.026)	(0.045)	(0.032)
Depressive symptoms PGS	− 0.067*	− 0.075	− 0.064	0.100***	0.111*	0.093**
	(0.029)	(0.047)	(0.037)	(0.026)	(0.046)	(0.032)
BMI PGS	− 0.211***	− 0.250***	− 0.185***	0.034	0.055	0.024
	(0.030)	(0.049)	(0.039)	(0.027)	(0.046)	(0.034)
Socioeconomic status						
Years of education	0.153***	0.164***	0.147***	− 0.081***	− 0.063**	− 0.095***
	(0.014)	(0.021)	(0.018)	(0.012)	(0.019)	(0.016)
Mother’s education	0.048***	0.058**	0.047**	− 0.020	− 0.026	− 0.015
	(0.012)	(0.020)	(0.015)	(0.011)	(0.018)	(0.013)
Father’s education	0.009	0.010	0.008	0.003	0.004	0.000
	(0.010)	(0.016)	(0.013)	(0.009)	(0.015)	(0.011)
Childhood SES (Ref. = Poor)						
Average	0.202**	0.204	0.189*	− 0.197***	− 0.151	− 0.212**
	(0.067)	(0.107)	(0.087)	(0.059)	(0.099)	(0.073)
Well off	0.184	0.052	0.267	− 0.044	0.076	− 0.102
	(0.114)	(0.182)	(0.147)	(0.099)	(0.168)	(0.123)
Varied or missing	0.287	0.563	0.101	− 0.355	− 0.335	− 0.381
	(0.232)	(0.387)	(0.294)	(0.223)	(0.402)	(0.268)
Female	0.087			0.331***		
	(0.055)			(0.051)		
Birth year	− 0.041***	− 0.038**	− 0.045***	0.014	− 0.010	0.025**
	(0.008)	(0.014)	(0.010)	(0.007)	(0.014)	(0.009)
Birth cohort (Ref. = Cohorts 0–3; < 1924 to 1947)						
Cohorts 4 and 5: 1948 to 1959	− 0.293**	− 0.260	− 0.304**	0.348***	0.691***	0.190
	(0.096)	(0.172)	(0.117)	(0.086)	(0.159)	(0.104)
Cohorts 6 and 7: 1960 to 1971	− 0.341*	− 0.328	− 0.317	0.381**	0.906***	0.127
	(0.161)	(0.284)	(0.197)	(0.143)	(0.268)	(0.172)
Age difference between outcome measured and age 50	− 0.054***	− 0.049***	− 0.057***	0.010	− 0.005	0.017
	(0.008)	(0.013)	(0.010)	(0.008)	(0.013)	(0.009)
/cut1	− 81.264***	− 74.651**	− 88.257***			
	(15.129)	(27.079)	(18.496)			
/cut2	− 79.661***	− 73.112**	− 86.612***			
	(15.128)	(27.077)	(18.495)			
/cut3	− 77.983***	− 71.325**	− 84.993***			
	(15.126)	(27.075)	(18.493)			
/cut4	− 76.292***	− 69.649*	− 83.279***			
	(15.125)	(27.073)	(18.490)			
/lnalpha				0.453***	0.502***	0.400***
				(0.044)	(0.078)	(0.054)
Constant				− 25.809	19.906	− 47.573**
				(14.181)	(26.669)	(17.122)
Observations	4899	1962	2937	4899	1962	2937
Pseudo R-squared	0.0407	0.0509	0.0380	0.0215	0.0252	0.0178
(− 2)Log-likelihood	12,750	5042	7672	12,958	4594	8326
Chi-squared	540.4	270.4	302.7	285.1	118.9	150.9

Standard errors in parentheses.

PGS, Polygenic score. Childhood SES: childhood socioeconomic status. Top ten PCs are controlled in the models.

^a^Interaction term between gender and partnership trajectory cluster is significant at *p* < 0.05.

***p < 0.001; **p < 0.01; *p < 0.05.

Several polygenic scores are associated with self‑rated health. A higher well‑being PGS is linked to better health in the pooled sample ($\beta = 0.10, SE = 0.03, p = 0.001$) and among women ($\beta = 1.31, SE = 0.04, p < 0.001$). Higher depressive symptoms PGS values are associated with poorer health in the pooled sample ($\beta = - 0.07, SE = 0.03, p = 0.021$), while a higher BMI PGS is linked to poorer health in all models ($Pooled: \beta = - 0.21, SE = 0.03, p < 0.001$; $Male: \beta = - 0.251, SE = 0.05, p < 0.001$; $Female: \beta = - 0.19, SE = 0.04, p < 0.001$).

Socioeconomic factors are also strongly related to self‑rated health. Years of education is positively associated with health for both men ($\beta = 1.16, SE = 0.02, p < 0.001$) and women ($\beta = 1.15, SE = 0.02, p < 0.001$). Mother’s education is also positively associated with health in the pooled sample and among men ($\beta = 0.06, SE = 0.02, p = 0.003$) and women ($\beta = 0.05, SE = 0.02, p = 0.002$). Respondents who reported average childhood SES (relative to poor) report better health in the pooled sample ($\beta = 0.20, SE = 0.07, p = 0.003$) and among women ($\beta = 0.19, SE = 0.09, p = 0.030$).

Figure 3 also shows that the coefficient for the educational attainment PGS is initially positive when only PGSs and partnership clusters are included. After socioeconomic status variables (SES) and controls are added, the coefficient becomes weaker, consistent with the close relationship between education and this PGS. In contrast, the coefficients for the well‑being and depressive symptoms PGSs remain largely unchanged across models.

#### CESD score

**Fig. 4:**
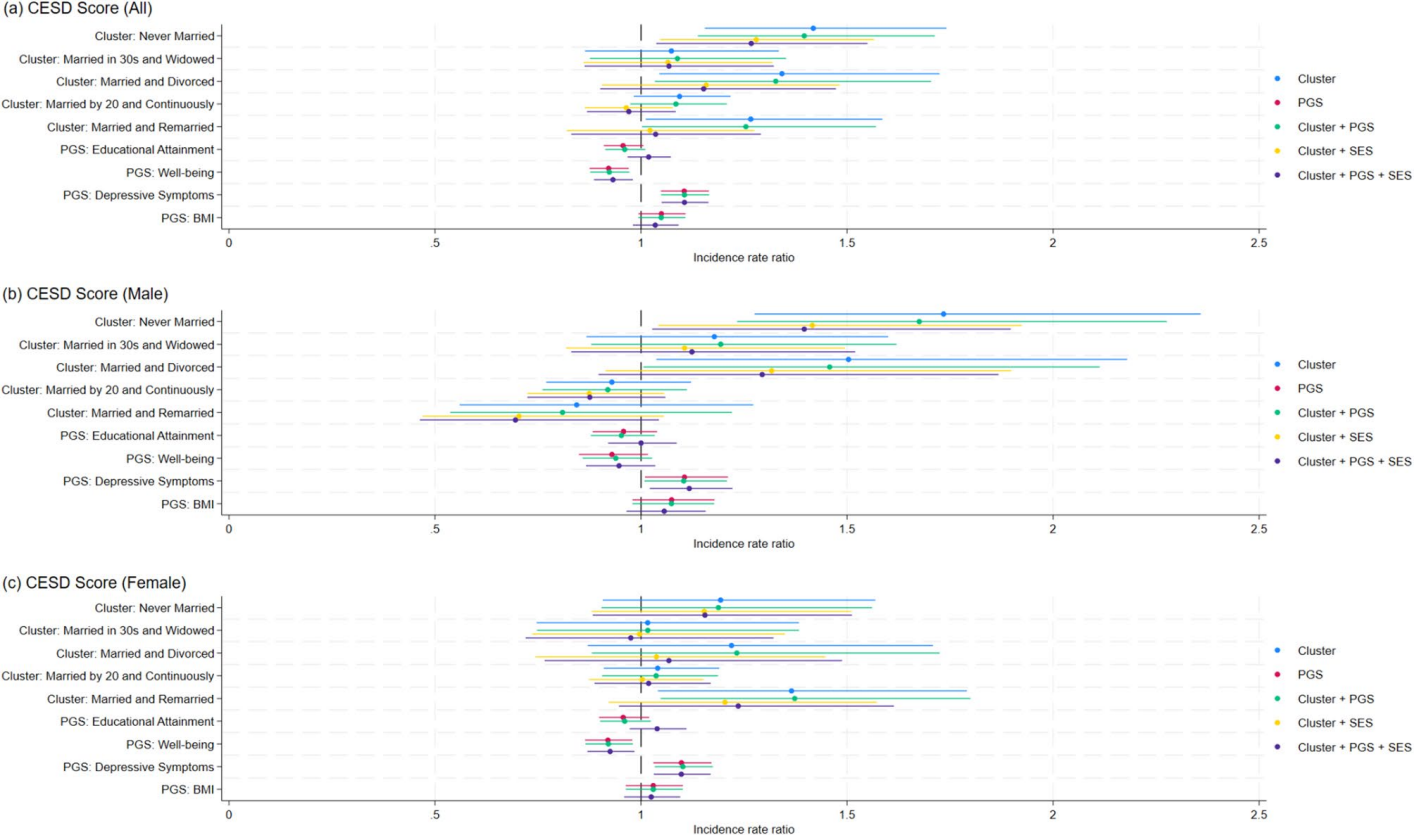
Incidence rate ratios for CESD score associated with partnership trajectories and polygenic scores (overall and by gender). Incidence rate ratios and 95% confidence intervals from negative binomial regression models predicting CESD score. Results are shown for the pooled sample (**a**), men (**b**), and women (**c**). Each set of points represents estimates from models including different combinations of predictors: partnership trajectory clusters only, polygenic scores (PGS) only, clusters plus PGS, clusters plus socioeconomic status (SES) and control variables, and clusters plus PGS, SES, and controls. The reference group for partnership clusters is “Married in 20s and Continuously Married”.

Figure 4 shows that when only partnership trajectories are included, respondents in the Never Married, Married and Divorced, and Married and Remarried clusters report higher CESD scores than those in the Married in 20s and Continuously Married cluster. The Never Married cluster shows the largest difference. Initially, the Married and Divorced cluster is significant only in men, and the Married and Remarried cluster is significant only in women. After PGSs are added, the coefficient for the Married and Divorced cluster becomes weaker, and the coefficient for the Married and Remarried cluster becomes insignificant. The associations weaken further after SES and control variables are included (see Supplementary Table S5).

In the fully adjusted model (Table 3, Panel B), only the Never Married cluster ($\beta = 0.24, SE = 0.10, p = 0.021$) remains significantly associated with depressive symptoms. This association is particularly strong among men ($\beta = 0.33, SE = 0.16, p = 0.033$), whereas no trajectory cluster is significant among women.

Among the polygenic scores, a higher well‑being PGS is associated with fewer depressive symptoms in the pooled sample ($\beta = - 0.07, SE = 0.03, p = 0.006$) and among women ($\beta = - 0.08, SE = 0.03, p = 0.014$). In contrast, a higher depressive symptoms PGS is associated with more depressive symptoms in the pooled sample ($\beta = 0.10, SE = 0.03, p < 0.001$), as well as among men ($\beta = 0.11, SE = 0.05, p = 0.016$) and women ($\beta = 0.09, SE = 0.03, p = 0.004$) separately. As shown in Fig. 4, the associations for the well‑being PGS and depressive symptoms PGS remain largely unchanged across model specifications.

Socioeconomic factors are also related to CESD scores. Years of education is negatively associated with depressive symptoms in the pooled sample ($\beta = - 0.08, SE = 0.01, p < 0.001$) and for both men ($\beta = - 0.06, SE = 0.02, p = 0.001$) and women ($\beta = - 0.10, SE = 0.02, p < 0.001$). Respondents with average childhood SES (compared with poor) report lower CESD scores in the pooled sample ($\beta = - 0.20, SE = 0.06, p = 0.001$) and in women ($\beta = - 0.21, SE = 0.07, p = 0.004$).

Overall, partnership trajectories, socioeconomic background, and polygenic scores are all associated with self‑rated health and depressive symptoms. Trajectories involving divorce or remaining unmarried are associated with poorer outcomes compared to the reference group (i.e., the Married in the 20s and Continuously Married cluster). The well‑being, depressive symptoms, and BMI PGSs also contribute to variation in both self-rated health and CESD score. Additionally, respondent education shows relatively consistent associations with both outcomes, while socioeconomic status variables explain the association between educational attainment PGS and self-rated health.

### Gender differences in associations of partnership trajectories with health outcomes

The analyses above indicate slight gender differences in the associations between partnership trajectories and health outcomes after accounting for PGSs and socioeconomic background. In the fully adjusted models, only women in the Married and Divorced cluster report poorer self‑rated health than the reference group, whereas only men in the Never Married cluster report higher CESD scores. All other associations between clusters and health outcomes diminish once socioeconomic background is considered.

To formally test gender differences, we add interaction terms between gender and trajectory clusters to the models predicting health outcomes (see Supplementary Table S6). Only one significant interaction is found. Women in the Married and Remarried cluster have significantly higher CESD scores than their male counterparts ($\beta = 0.55, SE = 0.24, p = 0.023$).

**Fig. 5:**
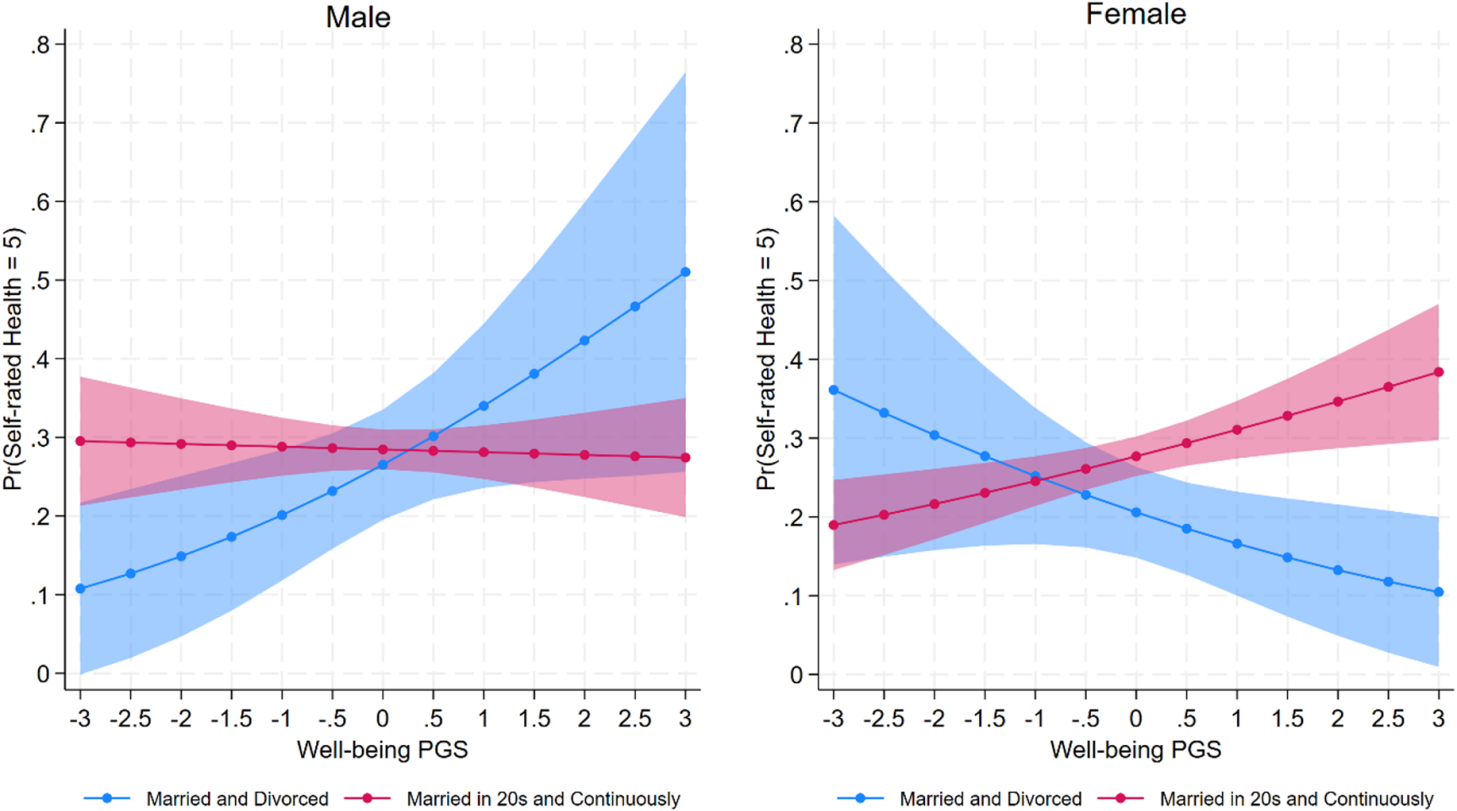
Interaction between well-being polygenic score and partnership trajectory cluster in predicting probability of excellent Self-rated Health, by gender. Predicted probabilities of reporting excellent Self-rated Health (category = 5) from ordered logit models including an interaction between well-being polygenic score (PGS) and partnership trajectory cluster, shown separately for men and women. Results are displayed for the “Married and Divorced” and “Married in 20s and Continuously” clusters. The interaction between well-being PGS and partnership trajectory is significant in opposite directions for men and women, and the three-way interaction with gender is significant.

Exploratory analyses test three‑way interactions between gender, trajectory cluster, and PGSs. Among all tests, only one significant result is observed: the association between the Married and Divorced cluster and self‑rated health varies by both gender and the well‑being PGS ($\beta = - 0.85, SE = 0.26, p = 0.001$; see Fig. 5). As the well-being PGS increases, the relationship between being in the Married and Divorced cluster and self-rated health becomes more negative for women but more positive for men. This finding suggests that partnership trajectories may have different implications for men and women depending on genetic predispositions related to well‑being.

## Discussion

Using nationally representative data from the U.S. Health and Retirement Study (HRS) combined with polygenic scores (PGSs) and socioeconomic measures, this study examined how partnership trajectories from ages 15 to 50 were related to self-rated health and depressive symptoms in later life. Six distinct trajectories were identified through sequence and cluster analysis. Respondents in long-term, continuous unions reported better health and fewer depressive symptoms than those who never married or, among women, experienced divorce. These differences remained after adjusting for PGSs and socioeconomic background, suggesting that the health effects of partnership histories are not fully explained by social or genetic selection processes.

Our findings demonstrate that both social and genetic factors are associated with partnership trajectories. Higher respondent education, parental education, and childhood socioeconomic advantage were linked to a greater likelihood of entering and sustaining long term unions, consistent with previous evidence that social advantage shapes marriage formation and stability^16,17,44,45^. Among the polygenic scores, the educational attainment PGS showed the most consistent associations. In the pooled sample, it was linked to a lower likelihood of being in clusters involving early marriage or remarriage. In gender stratified models, it was associated with both early marriage and divorce for women, but only with remarriage for men. Other PGSs showed more specific associations. For men, a higher well-being PGS was related to a lower likelihood of remaining never married, while higher depressive symptoms and BMI PGSs were related to a greater likelihood of belonging to the Married by 20 and Continuously cluster. For women, the associations of these PGSs with partnership trajectories were less consistent. These patterns align with findings from Jørgensen et al.^28^, who reported that polygenic indices for traits such as depression and well-being predicted partnership dissolution, with many associations persisting after accounting for shared family background. Overall, the results indicate that genetic predispositions contribute to relationship behaviors, while socioeconomic background remains a central factor shaping long term partnership stability.

Partnership trajectories were also associated with self-rated health in later life. Respondents in long term continuous unions reported better health than those who never married or experienced divorce, even after accounting for polygenic scores. In fully adjusted models where social and genetic factors were controlled, individuals in the Never Married cluster had poorer self-rated health, and among women, those in the Married and Divorced cluster also reported poorer health compared with those who married in their twenties and remained continuously married. These findings are consistent with cumulative advantage and disadvantage theory, which emphasizes that advantages and disadvantages accumulate across the life course to shape health in later life^14^. Stable partnerships may promote better health by providing sustained emotional support, pooled resources, and reinforcement of health-related behaviors^5,8,34^. In contrast, partnership disruptions can produce stress, financial strain, and social isolation, which have been linked to poorer physical and mental health outcomes^1,11,37,38^. In addition to partnership trajectories, higher well-being polygenic scores and lower depressive symptoms and BMI polygenic scores were associated with better self-rated health, while respondents with greater educational attainment, higher maternal education, and average (but not high) childhood socioeconomic backgrounds also reported better health.

For depressive symptoms, the associations with partnership trajectories were similar to those for self-rated health. In the fully adjusted models, respondents in the Never Married cluster reported more depressive symptoms than those in long term continuous unions, and this association was particularly strong among men. No trajectory clusters were significant for women. These findings highlight the particular disadvantage of never marrying for men’s psychological well-being, consistent with research showing that men tend to receive greater emotional and caregiving support from spouses^5,34^. The absence of significant effects among women may reflect the availability of other sources of social support as well as the greater caregiving responsibilities and financial strain women often experience after divorce, which could offset potential benefits of marriage^1,35,36^. Beyond partnership history, higher well-being polygenic scores and lower depressive symptoms polygenic scores were associated with fewer depressive symptoms, and individuals with more education and average (but not high) childhood socioeconomic backgrounds also reported lower CESD scores. These patterns underscore that both genetic predispositions and socioeconomic resources contribute to health outcomes in later life, with partnership history representing a life course pathway through which these factors operate.

Including genetic information allowed us to evaluate whether the apparent health benefits of marriage reflect selection rather than protection. Prior research shows that individuals who marry and remain married are more socially and biologically advantaged before marriage, suggesting a role for selection^44,46,47^. For example, Gueltzow et al.^29^ found that genetic risk for depression is linked to both partnership status and antidepressant use, indicating that genetic predispositions influence both union formation and mental health. Our findings extend this work by showing that although polygenic scores are associated with both partnership trajectories and health outcomes, they do not fully account for the associations between partnership histories and later-life health. This suggests that relationship experiences exert lasting effects beyond what can be explained by genetic selection alone.

To assess the robustness of our findings, we conducted several additional analyses. First, we examined whether the educational attainment PGS captures only genetic components related to education or whether it also reflects cognitive ability. We reran the models replacing the educational attainment PGS with the general cognition PGS (Supplementary Tables S7–S9). The results were nearly identical to the main findings, with only slight differences in coefficient sizes, suggesting that the educational attainment PGS and the general cognition PGS operate similarly in predicting partnership trajectories and health outcomes.

Second, we examined whether the clustering method influenced our conclusions by deriving partnership trajectory clusters using latent class analysis (LCA) based on partnership states measured at 5‑year intervals from ages 15 to 50 (Supplement Tables S10–S12). Although the clusters identified by LCA were broadly comparable to those from sequence analysis, key differences emerged because LCA requires selecting a limited set of measurement points. This may obscure information about the timing, duration, and sequencing of transitions between selected ages. In addition, latent class analysis assumes that, once individuals are assigned to a latent class, their observed partnership states at different time points are statistically independent of one another^48^. This assumption may be unrealistic for processes like partnership histories, where earlier states strongly influence later ones. In contrast, sequence analysis incorporates complete life-course information, capturing the timing and ordering of all transitions and identifying clusters that more directly reflect observed trajectories. For these reasons, sequence analysis was selected as the primary method for this study.

Despite these methodological differences, several patterns were consistent across approaches. In both analyses, a higher educational attainment PGS was negatively associated with membership in the Remarried cluster. However, whereas the main analysis showed a negative association between the educational attainment PGS and the Married by 20 and Continuously cluster, the LCA‑based analysis found a positive association with the Married around 30 cluster. This divergence likely reflects differences in how clusters were defined by each method.

The associations between LCA‑derived clusters and later‑life well‑being were broadly similar to those observed in the main analysis. For self‑rated health, men in the Never Married cluster reported poorer health than those in long‑term continuous unions, and women in the Divorced Early and Remarried cluster also reported poorer health than those in the Married Continuously cluster. For CESD scores, men in the Never Married cluster and women in the Divorced Early and Remarried cluster had higher depressive symptoms compared with continuously married counterparts. These findings are consistent with the main results and reinforce the conclusion that remaining unmarried or experiencing repeated disruptions is associated with poorer physical and mental health. At the same time, the modest differences in associations underscore how methodological choices in clustering can influence specific parameter estimates.

Third, to address the discrepancies in N across the construction and estimation samples (N = 9509 to build trajectories, N = 4922 to predict trajectory cluster, and N = 4899 to predict health outcomes) and to assess whether dropping cases affected the results, we implemented inverse probability weighting (IPW) (Supplementary Tables S13–S14). For each stage, we fit a logistic regression predicting inclusion in the corresponding analytic sample using birth year, birth cohort, and self-reported gender. Other covariates were excluded from the weight model because of substantial missingness. Each included respondent received an inverse probability weight defined as one divided by their predicted probability of inclusion, normalized to have a mean of one. We then re-estimated the multinomial logit for clusters and the ordered-logit (self-rated health) and negative-binomial (CESD) models using these weights.

The weighted estimates closely matched the originals, with a few shifts across conventional thresholds. The educational-attainment PGS became significant for being in the Married in 30s and Widowed cluster (versus Married in 20s and Continuously Married). The same PGS showed a weaker association (*p* < 0.10) for the Married by 20 and Continuously Married cluster in the pooled model and for the Married and Divorced cluster among women. Varied or missing childhood SES became highly significant for men in the Married and Remarried cluster. For health outcomes, Never Married became a significant negative correlate of men’s self-rated health. These limited changes suggest modest sensitivity to selection on birth year, cohort, and gender. However, the reduction from 9509 to 4922 and 4899 is largely due to missingness in key covariates (e.g., genetic measures, parental education), and IPW cannot correct bias from casewise deletion on variables not included in the weight model. In addition, other factors such as differential consent to genotyping, survival to follow-up, and item nonresponse may also contribute to case loss and small differences across models. Although these procedures cannot address all sources of selection, they increase confidence that our findings are not sensitive to selection on the observed factors used to construct the weights.

Finally, we analyzed data for non-Hispanic Black respondents (Supplementary Tables S15–S18). Several associations differed from those observed among non-Hispanic Whites. For instance, Blacks with higher well-being PGS were more likely to be in the Married in 30s and Widowed cluster, whereas those with higher BMI PGS were less likely to be in the Married and Divorced cluster. In models predicting self-rated health, initial associations for the Never Married and Married in 30s and Widowed clusters, as well as for the educational attainment PGS, became nonsignificant after adjusting for socioeconomic background. Similar attenuation was observed for CESD scores. These results likely reflect both the smaller sample size for non-Hispanic Black respondents and the reduced predictive validity of PGSs developed from European ancestry GWAS when applied to other ancestry groups. Future research with larger and more diverse genomic datasets is necessary to clarify the links between partnership trajectories, genetic predispositions, and well-being among African Americans.

By combining sequence analysis with molecular genetic data, this study provides new evidence on how social and genetic factors jointly shape partnership trajectories and their long term effects. Continuous unions were linked to better self-rated health and fewer depressive symptoms. Never marrying was associated with poorer health and more depressive symptoms, with the latter effect especially pronounced for men, and divorce was related to poorer health for women. These findings support prior research showing that both early life socioeconomic advantage and genetic predispositions influence the formation and maintenance of stable partnerships, which in turn contribute to disparities in health and psychological well-being across adulthood and into later life^1,4,49^.

## Limitations of this study

This study has several limitations that should be considered when interpreting the findings. First, the partnership histories were collected retrospectively, which may introduce recall error. Second, the analysis focused only on legal marriage histories and did not account for cohabiting or other nonmarital partnerships, which can also affect health outcomes^50^. Third, self-rated health and CESD scores captured only two dimensions of health and may not reflect broader aspects of physical and mental health. Fourth, sequence dissimilarities were computed with the optimal matching (OM) method using transition-rate substitution costs and unit insertion and deletion costs. Under this specification, two otherwise similar trajectories that begin marriage at different ages would incur higher alignment costs because OM inserts or deletes years to align the long marriage spells. As a result, OM distances are more sensitive to differences in the timing and duration of marriage, and clusters may place greater emphasis on marriage timing and union stability. Alternative distance definitions or cost settings could reassign some respondents and slightly alter cluster sizes. Fifth, sequence clustering requires analytic decisions regarding state definitions and the number of clusters to retain, which may have influenced the identified trajectory patterns^51^. Finally, the polygenic scores were derived primarily from genome wide association studies of European ancestry populations, which limits their predictive validity for other ancestry groups^52^. Future research should use prospective data and more diverse genetic datasets to better understand how social and genetic factors interact to shape partnership trajectories and their consequences over the life course.

## Conclusions

Both social and genetic factors influenced long-term partnership trajectories, and these trajectories remained strongly related to self-rated health and depressive symptoms in later life. Our results show that continuous unions are linked to better outcomes, whereas never marrying or experiencing divorce is associated with poorer outcomes. Divorced women have poorer self-rated health, whereas men who never marry report more depressive symptoms. Combining molecular genetic data with life course approaches provides new evidence on how structural inequalities and individual predispositions together shape health disparities as people age.

## Data, measures, and methods

### Data source

This study uses data from the Health and Retirement Study (HRS) to address the research questions outlined above. The HRS (http://hrsonline.isr.umich.edu/) is a nationally representative longitudinal survey of U.S. adults over the age of 50. Sponsored by the National Institute on Aging (NIA) and conducted by the Institute for Social Research (ISR) at the University of Michigan, the survey has been collected biennially since 1992. The HRS focuses on social, economic, and health-related factors associated with aging and retirement.

For this analysis, we draw on HRS survey waves from 1992 through 2020^53^, together with the 2015–2017 Life History Mail Survey (LHMS)^54^, genetic data collected in 2006, 2008, and 2012^55^, and the Childhood Family and Childhood Health Aggregated Data from 1992–2016^56^, to cover respondents’ partnership histories from ages 15 to 50 and their health outcomes thereafter. Although the analytic partnership sequences span ages 15 to 50, the sample includes respondents born between 1914 and 1968, allowing prediction of health outcomes measured after age 50.

To construct partnership trajectories, this study uses respondents’ self-reported relationship histories from the 2015–2017 Cross-Wave Life History Mail Survey (LHMS): Harmonized and Aggregated Public Data Resource^54^. The LHMS includes retrospective data on education, employment, partnership history, health, and other major life events. Data collection took place in Fall 2015, Spring 2017, and Fall 2017, with partnership history questions asked in 2017. As a result, the partnership histories used in this study extend through to 2017. The dataset includes 11,761 respondents, and this study uses partnership histories from age 15 to age 50 to construct partnership sequences and categorize trajectories.

In addition to survey data, HRS collected genetic data through saliva specimens in 2006, 2008, and 2012. More than 19,000 samples were genotyped using the Illumina HumanOmni2.5 BeadChips at the Center for Inherited Disease Research, and 15,000 samples passed quality control at the Genetics Coordinating Center of the University of Washington. This study uses HRS Polygenic Scores—Release 4^55^, which includes polygenic scores (PGSs) constructed separately for respondents of European ancestry (non-Hispanic White; N = 12,090) and African ancestry (N = 3100) in the original release file. Because PGSs derived from European-based genome-wide association studies have limited predictive validity in non-European ancestry populations, the primary analyses in this study are restricted to respondents of European ancestry. Analyses involving non-Hispanic Black respondents are included in the supplementary file (Supplementary Tables S15–S18) for reference.

To account for childhood family socioeconomic background, this study uses the Childhood Family and Childhood Health Aggregated Data^56^. This dataset compiles retrospective reports from respondents across HRS waves from 1992 to 2016. A total of 38,654 respondents are included in this dataset, providing measures of childhood socioeconomic conditions.

We constructed partnership trajectories from the Life History Mail Survey using respondents’ reported years of marriage and dissolution and reasons for dissolution, and defined the trajectory-construction sample as respondents with valid partnership histories from ages 15 to 50 (N = 9509). For analyses involving genetic data, we restrict to respondents of European ancestry with available polygenic scores and intersect this set with the trajectory-construction sample (N = 5493); adding respondent years of education yields N = 5478; and adding mother’s and father’s education results in an analytic sample of non-Hispanic White respondents (N = 4922). In the first stage of analysis, which estimates predictors of trajectory cluster membership, we analyze respondents with clusters, polygenic scores, and complete covariates (N = 4922). In the second stage, which estimates associations between clusters and later-life health, we apply the same criteria and further require nonmissing self-rated health and CESD depressive symptoms (final N = 4899).

### Measures

This study involves two main analytic stages. The first stage uses sequence analysis to classify partnership trajectories into clusters and to examine the social and genetic factors associated with these patterns. The second stage uses these clusters as key independent variables in regression analyses to evaluate their associations with later-life health outcomes, corresponding to the second and third research questions. Detailed analytical procedures are provided in Analytical strategies section.

In both stages, polygenic scores (PGSs) are incorporated to account for genetic predispositions. A PGS summarizes the combined effects of multiple genetic variants (single-nucleotide polymorphisms, or SNPs) on an individual’s likelihood of exhibiting a particular trait. In HRS, PGSs are constructed using all available SNPs that overlap between genome-wide association studies (GWAS) of specific traits and the HRS genotype data. GWAS results provide weights (beta estimates or odds ratios) for each SNP, indicating the direction and magnitude of association with the trait. If a beta value is negative, the reference allele is adjusted accordingly so that all effect sizes are positive. The PGS for an individual is calculated using the equation: $PGS_{i} = \sum\nolimits_{j = 1}^{J} {W_{j} \times G_{ij} }$, where i represents an individual, j denotes the SNP, $W_{j}$ is the effect size for SNP j, and $G_{ij}$ is the number of reference alleles (0, 1, or 2) that the individual carries at SNP j.

Because this study focuses on non‑Hispanic White respondents for the main analysis, PGSs derived from GWAS results based on European ancestry samples are used exclusively. We included PGSs for educational attainment^23^, BMI^57^, well‑being, and depressive symptoms^26^, as these traits have been associated with both partnership formation or stability and adult health outcomes^28,33^. For robustness, a PGS for general cognition^58^ is also included to compare with the educational attainment PGS (see Supplementary Tables S7–S9).

The health outcome variables in the second stage of analysis capture two dimensions: physical health and mental health. Physical health is measured using self-rated health. Respondents were asked to rate their current health on a five-point Likert scale (1 = excellent to 5 = poor). For ease of interpretation, the scale is reverse-coded so that 1 = poor and 5 = excellent. Mental health is measured using the CESD score, a widely used index of depressive symptoms^53^. The score is calculated as the sum of six negative indicators (e.g., feeling depressed, everything is an effort, restless sleep) and the reverse-coded values of two positive indicators (e.g., feeling happy, enjoying life). The CESD score ranges from 0 to 8, with higher values indicating more depressive symptoms.

To align with the sequence analysis timeframe (ending at age 50), self-rated health and CESD scores are taken from the first available survey wave after respondents turned 50. For example, if a respondent turned 50 in 2013, health measures are drawn from the 2014 HRS wave. To account for variation in the timing of measurement, the number of years between age 50 and the health assessment is included as a control variable.

Several variables are included to account for early-life socioeconomic background (SES). These include father’s and mother’s years of education and the respondent’s own years of schooling, which serve as indicators of socioeconomic advantage or disadvantage. Childhood socioeconomic status is also measured using a retrospective self-assessment of the family’s financial situation between birth and age 16. Respondents were asked: “Now think about your family when you were growing up, from birth to age 16. Would you say your family during that time was pretty well off financially, about average, or poor?” Response options include: don’t know, it varied, poor, about average, and pretty well off financially. Given their ambiguity, don’t know and it varied are combined into a single category, and the variable is treated as categorical in the analysis.

Other control variables include self-reported gender, birth year, birth cohort, and the top ten principal components (PCs) for population stratification. As recommended by HRS, the top ten genetic PCs of genetic ancestry are included to adjust for subtle population structure within the European ancestry sample and to reduce potential confounding from population stratification^59^.

Given that social norms around marriage and partnership vary across cohorts, HRS-defined birth cohorts are used to control for cohort effects. The original HRS includes seven birth cohorts: AHEAD (born before 1924), Children of the Depression (CODA; 1924–1930), HRS (1931–1941), War Babies (1942–1947), Early Baby Boomers (EBB; 1948–1953), Mid Baby Boomers (MBB; 1954–1959), and Late Baby Boomers (LBB; 1960–1965). For analysis, these are grouped into three broader categories to ensure sufficient cell sizes: those born before 1948, those born between 1948 and 1959, and those born in 1960 or later.

### Analytical strategies

The analysis proceeds in two stages. In the first stage, we use sequence analysis to classify respondents’ partnership histories from age 15 to 50, constructing partnership trajectory categories in the trajectory-construction sample (N = 9509). Sequence analysis is a holistic life course approach that accounts for the timing, duration, and ordering of transitions, allowing for the identification of typical trajectories^51^. This method recognizes that relationship histories unfold as interconnected processes rather than isolated events.

Respondents reported the start and end years of their partnerships. Based on this information, each respondent was assigned to one of five states in each year: not married, married, remarried, divorced, or widowed. Missing values between two known observations were filled by carrying forward the most recent observed state. For example, if a respondent was married in 1985 and the next valid record was in 1988, and no divorce or widowhood was reported in between, the intervening years (1986–1987) were imputed as “married”. This imputation procedure was combined with additional logic corrections to ensure that partnership sequences were consistent with plausible life course transitions. For example, direct transitions from “married” to “not married” were disallowed, as a married individual can only transition to either “divorced” or “widowed”. Similarly, “not married” to “divorced” or “widowed” transitions were permitted only if a prior marriage was recorded.

To quantify differences between individuals’ partnership sequences, we apply optimal matching (OM), which identifies the minimum edit cost required to transform one sequence into another^60^. The cost reflects how dissimilar two life-course trajectories are in both timing and ordering of transitions. It is determined by the number and type of operations needed to align one sequence with another: substitutions, where one partnership state is replaced by another; insertions, where a state is added; and deletions, where a state is removed. Substitution costs are derived empirically from observed transition probabilities following Piccarreta and Billari^61^, so that common transitions such as marriage followed by divorce incur lower costs, whereas rare transitions such as widowhood followed by remarriage incur higher costs. Insertion and deletion costs are fixed at 1, which lets the algorithm shift sequences slightly by adding or removing a small number of positions when timing differs rather than substituting many mismatched states. This preserves the ordering of states and the length of consecutive years spent in each state. In many sequences, marriage is followed by a long continuous period in the married state, so differences in the age at first marriage can influence the alignment cost when these long periods do not line up. Differences involving divorce, remarriage, and widowhood also contribute to distances through their empirically derived substitution costs. The resulting dissimilarity matrix summarizes how partnership trajectories differ across ages 15 to 50 and serves as the basis for clustering.

Once the dissimilarity matrix is constructed, we use Ward’s linkage algorithm^62^ to identify clusters of similar partnership trajectories. This hierarchical clustering method minimizes within-group variance and yields distinct trajectory groups that capture common patterns of partnership experiences over the life course. These trajectory clusters serve as the dependent variable in multinomial logit models that assess the associations between social and genetic factors and partnership patterns.

In the second stage, we evaluate the relationship between partnership trajectories and health outcomes using ordered logit models for self-rated health and negative binomial models for CESD scores, given the overdispersion of the latter. These models address the second research question by testing whether partnership trajectories and genetic predispositions are associated with later-life physical and mental health. We adjust for genetic and social background factors in the models to account for selection into partnership histories and health. Covariates include PGSs for educational attainment, well-being, depressive symptoms, and BMI; early-life socioeconomic background captured by parents’ education and retrospective childhood SES; respondent years of education; self-reported gender; birth year and birth cohort; the years between age 50 and the health assessment; and the top ten genetic principal components. PGSs are included in the second stage to reduce genetic confounding that may jointly influence trajectories and health, and social factors are included to assess whether genetic predispositions and social background each show independent associations with health, net of the other.

Analyses are also stratified by gender to examine gender differences in the associations between partnership trajectories, genetic influences, and health outcomes. Primary estimates are reported for the pooled sample combining men and women, with gender-stratified results presented alongside.

Analyses proceed as follows. Descriptive sequence and cluster analyses draw on the trajectory-construction sample (N = 9509). In the first stage, we estimate predictors of trajectory-cluster membership using respondents with constructed clusters, PGSs, and complete covariates (N = 4922). In the second stage, we estimate associations between trajectory clusters and later-life health using the same criteria and requiring observed self-rated health and CESD depressive symptoms (N = 4899). Outcome-specific Ns are reported in the tables, and the lower N in the second stage reflects missing outcomes. Parallel summary statistics and results for non-Hispanic Black respondents are reported in the supplementary file (final N = 749).

## Supplementary Information

Below is the link to the electronic supplementary material.

Supplementary Material 1

## Data Availability

The data used in this study are publicly available from the Health and Retirement Study (https://hrs.isr.umich.edu) upon registration and completion of a data use agreement.

## References

[1] Hughes, M. E. & Waite, L. J. Marital biography and health at mid-life. *J. Health Soc. Behav.* **50**, 344–358 (2009). 19711810 10.1177/002214650905000307 PMC3148098

[2] Mäki, M., Hägglund, A. E., Rotkirch, A., Kulathinal, S. & Myrskylä, M. Stable marital histories predict happiness and health across educational groups. *Eur. J. Popul.* **41**, 12 (2025). 40358790 10.1007/s10680-025-09733-x PMC12075088

[3] Tambellini, E., Danielsbacka, M. & Rotkirch, A. Both partnership history and current relationship quality are associated with life satisfaction in old age. *Res. Aging* **47**, 193–209 (2025). 39688541 10.1177/01640275241309255

[4] Umberson, D. & Thomeer, M. B. Family matters: Research on family ties and health, 2010 to 2020. *J. Marriage Fam.* **82**, 404–419 (2020). 33867573 10.1111/jomf.12640 PMC8048175

[5] Waite, L. J. Does marriage matter?. *Demography* **32**, 483–507 (1995). 8925942

[6] Zhai, X. et al. Association and causal mediation between marital status and depression in seven countries. *Nat. Hum. Behav.* **8**, 2392–2405 (2024). 39496771 10.1038/s41562-024-02033-0

[7] Hanus, S. L., Simons, L. G., Lei, M.-K., Cobb, R. J. & Simons, R. L. Romantic relationship status, quality, and depressive symptoms among middle-aged and older black women. *J. Gerontol. Ser. B* **77**, 2126–2136 (2022). 10.1093/geronb/gbac016 PMC9683486 35091742

[8] Ross, C. E., Mirowsky, J. & Goldsteen, K. The impact of the family on health: The decade in review. *J. Marriage Fam.* **52**, 1059 (1990).

[9] Shrout, M. R. et al. Dyadic, biobehavioral, and sociocultural approaches to romantic relationships and health: Implications for research, practice, and policy. *Soc. Personal. Psychol. Compass* **18**, e12943 (2024).

[10] Umberson, D., Pudrovska, T. & Reczek, C. Parenthood, childlessness, and well-being: A life course perspective. *J. Marriage Fam.* **72**, 612–629 (2010). 21869847 10.1111/j.1741-3737.2010.00721.x PMC3159916

[11] Amato, P. R. The consequences of divorce for adults and children. *J. Marriage Fam.* **62**, 1269–1287 (2000).

[12] Jung, J. Partnership trajectories and their consequences over the life course. Evidence from the German LifE Study. *Adv. Life Course Res.* **55**, 100525 (2023). 36942643 10.1016/j.alcr.2022.100525

[13] Musick, K. & Bumpass, L. Reexamining the case for marriage: Union formation and changes in well-being. *J. Marriage Fam.* **74**, 1–18 (2012). 22611285 10.1111/j.1741-3737.2011.00873.x PMC3352182

[14] Dannefer, D. Cumulative advantage/disadvantage and the life course: Cross-fertilizing age and social science theory. *J. Gerontol. B. Psychol. Sci. Soc. Sci.* **58**, S327–S337 (2003). 14614120 10.1093/geronb/58.6.s327

[15] Elder, G. H. Time, human agency, and social change: Perspectives on the life course. *Soc. Psychol. Q.* **57**, 4 (1994).

[16] Fulda, B. E. The diversity in longitudinal partnership trajectories during the transition to adulthood: How is it related to individual characteristics and regional living conditions?. *Demogr. Res.* **35**, 1101–1134 (2016).

[17] Hiekel, N. & Vidal, S. Childhood family structure and complexity in partnership life courses. *Soc. Sci. Res.* **87**, 102400 (2020). 32279859 10.1016/j.ssresearch.2019.102400

[18] Sironi, M. The role of fertility and partnership history in later-life cognition. *Ageing Int*10.1007/s12126-022-09500-x (2022).

[19] Barban, N. et al. Genome-wide analysis identifies 12 loci influencing human reproductive behavior. *Nat. Genet.* **48**, 1462–1472 (2016). 27798627 10.1038/ng.3698 PMC5695684

[20] Mathieson, I. et al. Genome-wide analysis identifies genetic effects on reproductive success and ongoing natural selection at the FADS locus. *Nat. Hum. Behav.* **7**, 790–801 (2023). 36864135 10.1038/s41562-023-01528-6

[21] Mills, M. C. et al. Identification of 371 genetic variants for age at first sex and birth linked to externalising behaviour. *Nat. Hum. Behav.* **5**, 1717–1730 (2021). 34211149 10.1038/s41562-021-01135-3 PMC7612120

[22] Venkatesh, S. S. et al. Genome-wide analyses identify 25 infertility loci and relationships with reproductive traits across the allele frequency spectrum. *Nat. Genet.* **57**, 1107–1118 (2025). 40229599 10.1038/s41588-025-02156-8 PMC12081293

[23] Lee, J. J. et al. Gene discovery and polygenic prediction from a genome-wide association study of educational attainment in 1.1 million individuals. *Nat. Genet.* **50**, 1112–1121 (2018). 30038396 10.1038/s41588-018-0147-3 PMC6393768

[24] Okbay, A. et al. Polygenic prediction of educational attainment within and between families from genome-wide association analyses in 3 million individuals. *Nat. Genet.* **54**, 437–449 (2022). 35361970 10.1038/s41588-022-01016-z PMC9005349

[25] Adams, M. J. et al. Trans-ancestry genome-wide study of depression identifies 697 associations implicating cell types and pharmacotherapies. *Cell* **188**, 640-652.e9 (2025). 39814019 10.1016/j.cell.2024.12.002 PMC11829167

[26] Okbay, A. et al. Genetic variants associated with subjective well-being, depressive symptoms, and neuroticism identified through genome-wide analyses. *Nat. Genet.* **48**, 624–633 (2016). 27089181 10.1038/ng.3552 PMC4884152

[27] Turley, P. et al. Multi-trait analysis of genome-wide association summary statistics using MTAG. *Nat. Genet.* **50**, 229–237 (2018). 29292387 10.1038/s41588-017-0009-4 PMC5805593

[28] Jørgensen, R. E., Cheesman, R., Andreassen, O. A. & Lyngstad, T. H. The genetics of partnership dissolution. *Sociol. Sci.* **12**, 76–96 (2025).

[29] Gueltzow, M., Lahtinen, H., Bijlsma, M. J., Myrskylä, M. & Martikainen, P. Genetic propensity to depression and the role of partnership status. *Soc. Sci. Med.* **351**, 116992 (2024). 38772210 10.1016/j.socscimed.2024.116992

[30] Domingue, B. W., Liu, H., Okbay, A. & Belsky, D. W. Genetic heterogeneity in depressive symptoms following the death of a spouse: Polygenic score analysis of the US Health and Retirement Study. *Am. J. Psychiatry* **174**, 963–970 (2017). 28335623 10.1176/appi.ajp.2017.16111209 PMC5610918

[31] McErlean, K. The growth of education differentials in marital dissolution in the United States. *Demogr. Res.* **45**, 841 (2021). 35386929 10.4054/demres.2021.45.26 PMC8980992

[32] Musick, K., Brand, J. E. & Davis, D. Variation in the relationship between education and marriage: Marriage market mismatch?. *J. Marriage Fam.* **74**, 53–69 (2012). 22563132 10.1111/j.1741-3737.2011.00879.x PMC3340888

[33] Domingue, B. W., Fletcher, J., Conley, D. & Boardman, J. D. Genetic and educational assortative mating among US adults. *Proc. Natl. Acad. Sci.* **111**, 7996–8000 (2014). 24843128 10.1073/pnas.1321426111 PMC4050565

[34] Bird, C. E. & Rieker, P. P. *Gender and Health: The Effects of Constrained Choices and Social Policies* (Cambridge University Press, 2008).

[35] Pinquart, M. & Sörensen, S. Gender differences in caregiver stressors, social resources, and health: An updated meta-analysis. *J. Gerontol. B. Psychol. Sci. Soc. Sci.* **61**, P33–P45 (2006). 16399940 10.1093/geronb/61.1.p33

[36] Simon, R. W. Revisiting the relationships among gender, marital status, and mental health. *Am. J. Sociol.* **107**, 1065–1096 (2002). 10.1086/339225 12227382

[37] Zimmermann, O. & Hameister, N. Stable cohabitational unions increase quality of life: Retrospective analysis of partnership histories also reveals gender differences. *Demogr. Res.* **40**, 657–692 (2019).

[38] Umberson, D., Lin, Z. & Cha, H. Gender and social isolation across the life course. *J. Health Soc. Behav.* **63**, 319–335 (2022). 35856404 10.1177/00221465221109634 PMC10409601

[39] Idler, E. L. & Benyamini, Y. Self-rated health and mortality: A review of twenty-seven community studies. *J. Health Soc. Behav.* **38**, 21–37 (1997). 9097506

[40] Steptoe, A., Deaton, A. & Stone, A. A. Subjective wellbeing, health, and ageing. *Lancet* **385**, 640–648 (2015). 25468152 10.1016/S0140-6736(13)61489-0 PMC4339610

[41] Organisation for Economic Co-operation and Development (OECD) Group. *OECD Guidelines on Measuring Subjective Well-Being (2025 Update)* (OECD Publishing, Paris, 2025). 10.1787/9203632a-en.

[42] Radloff, L. S. The CES-D Scale: A self-report depression scale for research in the general population. *Appl. Psychol. Meas.* **1**, 385–401 (1977).

[43] Turvey, C. L., Wallace, R. B. & Herzog, R. A revised CES-D measure of depressive symptoms and a DSM-based measure of major depressive episodes in the elderly. *Int. Psychogeriatr.* **11**, 139–148 (1999). 11475428 10.1017/s1041610299005694

[44] Goldman, N. Marriage selection and mortality patterns: Inferences and fallacies. *Demography* **30**, 189–208 (1993). 8500636

[45] Johnson, D. R. & Wu, J. An empirical test of crisis, social selection, and role explanations of the relationship between marital disruption and psychological distress: A pooled time-series analysis of four-wave panel data. *J. Marriage Fam.* **64**, 211–224 (2002).

[46] Lipowicz, A. Some evidence for health-related marriage selection: Health-related marriage selection. *Am. J. Hum. Biol.* **26**, 747–752 (2014). 25065487 10.1002/ajhb.22588

[47] Lucas, R. E., Clark, A. E., Georgellis, Y. & Diener, E. Reexamining adaptation and the set point model of happiness: Reactions to changes in marital status. *J. Pers. Soc. Psychol.* **84**, 527–539 (2003). 12635914 10.1037//0022-3514.84.3.527

[48] Nylund-Gibson, K. & Ten Choi, A. Y. frequently asked questions about latent class analysis. *Transl. Issues Psychol. Sci.* **4**, 440 (2018).

[49] Carr, D. & Springer, K. W. Advances in families and health research in the 21st century. *J. Marriage Fam.* **72**, 743–761 (2010).

[50] Wright, M. R. & Brown, S. L. Psychological well-being among older adults: The role of partnership status: Psychological well-being among older adults. *J. Marriage Fam.* **79**, 833–849 (2017). 28626245 10.1111/jomf.12375 PMC5469370

[51] Abbott, A. Sequence analysis: New methods for old ideas. *Annu. Rev. Sociol.* **21**, 93–113 (1995).

[52] Ware, E. B. et al. *Heterogeneity in Polygenic Scores for Common Human Traits*. 10.1101/106062. (2017).

[53] Health and Retirement Study. RAND HRS Longitudinal File 2020 (V1) public use dataset. (2023).

[54] Health and Retirement Study. Cross-wave 2015-2017 Life History Mail Survey (LHMS): Harmonized and Aggregated Public Data Resource. (2021).

[55] Ware, E., Gard, A., Schmitz, L. & Faul, J. HRS Polygenic Scores—Release 4. (2020).

[56] Health and Retirement Study. Childhood Family and Childhood Health Aggregated Data: Version 2.0. (2020).

[57] Yengo, L. et al. Meta-analysis of genome-wide association studies for height and body mass index in ∼ 700000 individuals of European ancestry. *Hum. Mol. Genet.* **27**, 3641–3649 (2018). 30124842 10.1093/hmg/ddy271 PMC6488973

[58] Davies, G. et al. Genetic contributions to variation in general cognitive function: A meta-analysis of genome-wide association studies in the CHARGE consortium (N = 53 949). *Mol. Psychiatry* **20**, 183–192 (2015). 25644384 10.1038/mp.2014.188 PMC4356746

[59] Price, A. L. et al. Principal components analysis corrects for stratification in genome-wide association studies. *Nat. Genet.* **38**, 904–909 (2006). 16862161 10.1038/ng1847

[60] Abbott, A. & Hrycak, A. Measuring resemblance in sequence data: An optimal matching analysis of musicians’ careers. *Am. J. Sociol.* **96**, 144–185 (1990).

[61] Piccarreta, R. & Billari, F. C. Clustering work and family trajectories by using a divisive algorithm. *J. R. Stat. Soc. Ser. A Stat. Soc.* **170**, 1061–1078 (2007).

[62] Ward, J. H. Jr. Hierarchical grouping to optimize an objective function. *J. Am. Stat. Assoc.* **58**, 236–244 (1963).

